# Metagenomic insights into diazotrophic communities across Arctic glacier forefields

**DOI:** 10.1093/femsec/fiy114

**Published:** 2018-06-12

**Authors:** Maisie V Nash, Alexandre M Anesio, Gary Barker, Martyn Tranter, Gilda Varliero, Emiley A Eloe-Fadrosh, Torben Nielsen, Thomas Turpin-Jelfs, Liane G Benning, Patricia Sánchez-Baracaldo

**Affiliations:** 1 School of Geographical Sciences, University of Bristol, UK; 2 School of Life Sciences, University of Bristol, UK; 3 DOE Joint Genome Institute, 2800 Mitchell Drive, Walnut Creek, CA 94598, US; 4 GFZ German Research Centre for Geosciences, Telegrafenenberg, 14473 Potsdam, Germany; 5 School of Earth and Environment, University of Leeds, LS2 9JT, Leeds, UK; 6 Department of Earth Sciences, Free University of Berlin, Malteserstr, 74-100, Building A, 12249, Berlin, Germany

**Keywords:** nitrogen fixation, metagenomics, forefield, Arctic, diversity, diazotrophs

## Abstract

Microbial nitrogen fixation is crucial for building labile nitrogen stocks and facilitating higher plant colonisation in oligotrophic glacier forefield soils. Here, the diazotrophic bacterial community structure across four Arctic glacier forefields was investigated using metagenomic analysis. In total, 70 soil metagenomes were used for taxonomic interpretation based on 185 nitrogenase (nif) sequences, extracted from assembled contigs. The low number of recovered genes highlights the need for deeper sequencing in some diverse samples, to uncover the complete microbial populations. A key group of forefield diazotrophs, found throughout the forefields, was identified using a nifH phylogeny, associated with nifH Cluster I and III. Sequences related most closely to groups including Alphaproteobacteria, Betaproteobacteria, Cyanobacteria and Firmicutes. Using multiple nif genes in a Last Common Ancestor analysis revealed a diverse range of diazotrophs across the forefields. Key organisms identified across the forefields included *Nostoc*, *Geobacter*, *Polaromonas* and *Frankia*. Nitrogen fixers that are symbiotic with plants were also identified, through the presence of root associated diazotrophs, which fix nitrogen in return for reduced carbon. Additional nitrogen fixers identified in forefield soils were metabolically diverse, including fermentative and sulphur cycling bacteria, halophiles and anaerobes.

## Teaser

Diverse communities of nitrogen fixing bacteria are identified across Arctic glacier forefields, revealed using community genomic sequencing.

## INTRODUCTION

Arctic glaciers are undergoing fast retreat, exposing soils that have been locked under ice for thousands of years (Bradley, Singarayer and Anesio 2014). Microbial communities have been identified as the primary colonisers of these newly exposed soils (Schmidt *et al.*^2008^; Bradley *et al.*^2015^) and are important for building up initial carbon and nitrogen pools, enhancing soil stability through the release of exopolymeric substances, and mediating forefield soil pH (Sattin *et al.*^2009^; Schulz *et al.*^2013^; Bradley, Singarayer and Anesio 2014). However, there is a lack of coherent understanding on the diversity and biogeochemical importance of these bacterial communities in relation to nitrogen fixation (Brankatschk *et al.*^2011^). Bacterial nitrogen fixation uses the enzyme nitrogenase to convert atmospheric nitrogen (N_2_) into fixed ammonia (NH_3_) for biological uptake by non-diazotrophic organisms (Brill 1975). As nitrogen is a key nutrient for microbe and plant growth, nitrogen limited forefield soils may place restrictions on heterotroph colonisation, productivity and succession (Duc *et al.*2009a). Subsequently, diazotrohic organisms have been proposed as crucial facilitators of succession in newly exposed forefield soils (Knelman *et al.*^2012^). Nitrogen fixing Cyanobacteria have been identified as key in building these initial nitrogen stocks, and therefore expediting the establishment of heterotrophic organisms (Kaštovská *et al.*^2005^; Schmidt *et al.*^2008^; Duc *et al.*2009a).

Whilst the importance of early diazotrophs is evident, similarities and variations in the nitrogen-fixing communities across forefields, in terms of both diversity and phylogeny, have received limited attention. The majority of research to date has focused on understanding changes in nitrogen fixation within individual forefields, along transects or chronosequences of soil development (Duc *et al.*2009a; Brankatschk *et al.*^2011^). Thus far, the taxonomic diversity and abundance of the nifH gene has been shown to decrease with soil age and distance from the glacier terminus, in line with increasing fixed nitrogen in soils, and a reduced need for diazotrophy (Duc *et al.*2009a; Brankatschk *et al.*^2011^). The dominant diazotrohic community composition in forefields is likely to be influenced by factors such as soil physicochemical status, climate, topography, the establishment of plants and any disturbances, such as water flow pathways, which may elicit both similarities and differences in diazotrophy between sites (Hodkinson, Coulson and Webb 2003; Nicol *et al.*^2005^; Schütte *et al.*^2010^; Liu *et al.*^2012^). Furthermore, the current body of evidence surrounding microbial succession in forefields has a limited geographical range, with most studies conducted in the Damma Glacier forefield in Switzerland (Duc *et al.*2009a; Frey *et al*. 2010; Bernasconi *et al.*^2011^; Brankatschk *et al.*^2011^; Brunner *et al.*^2011^; Zumsteg *et al.*^2012^, ^2013^; Bradley *et al.*^2015^). Investigation across multiple glacier forefields is needed to fully explore similarities and differences between forefields in terms of diazotrophic community composition and their phylogenetic relations (Schütte *et al.*^2010^). This will help highlight the microbial community diversity involved in nitrogen fixation among glacier forefields.

Bacterial nitrogen fixation is encoded by clustered nitrogenase genes, typically through an enzyme containing an iron (Fe) cofactor and a molybdenum-iron (Mo-Fe) cofactor (Dixon and Kahn 2004). Overall, the abundance of bioavailable nitrogen controls the transcription of nitrogenase genes, whilst the variant of nitrogenase transcribed is regulated by the presence of molybdenum (Oda *et al.*^2005^; Teixeira *et al.*^2008^). In the absence of Mo, nitrogenase is transcribed with vanadium (Fe-V co-factor), or exclusively with iron (Fe-Fe cofactor) in the absence of both Mo and V (Raymond *et al.*^2004^; Teixeira *et al.*^2008^). These nitrogenases are in turn encoded by the nifHDK, vnfH-vnfDGK and anfHDGK operons (Dixon and Kahn 2004; Teixeira *et al.*^2008^). The phylogenetically conserved nifH gene can be used to classify bacterial diazotrophs into Clusters I-IV based on the nitrogenase (Chien and Zinder 1996). Cluster I covers the typical Mo nifH, whilst Cluster II covers the alternative vnfH and Cluster III generally includes a diverse range of anaerobic bacteria (Zehr *et al.*^2003^). Furthermore, Cluster IV contains organisms with ‘nif-like’ sequences, as opposed to conventional nif genes (Zehr *et al.*^2003^).

Previous research conducted on microbial succession in glacial forefields, including those on functional genes, has mostly focused on marker gene data, such as 16s rRNA and amplified nifH (Schmidt *et al.*^2008^; Brankatschk *et al.*^2011^; Rime, Hartmann and Brunner 2015). However, studies are now applying alternative methods, such as metagenomics, to study microbial communities (Wooley, Godzik and Friedberg 2010). This is because metagenomics provides gene sequences for the entire microbial community gene pool, rather than target sequences (Handelsman 2004; Daniel 2005). Thus, both microbial diversity and functional potential can be inferred using one approach (Wooley, Godzik and Friedberg 2010; Thomas, Gilbert and Meyer 2012). In order to maximise the quality of the output metagenome, the short DNA fragments from next generation sequencing should be assembled (Vázquez-Castellanos *et al.*^2014^). This generates longer continuous DNA reads (contigs), which provide more accurate functional and taxonomic annotations (Howe *et al.*^2014^; Vázquez-Castellanos *et al.*^2014^).

In this study, we investigated 70 soil metagenomes spanning transects and chronosequences across four Arctic forefields in N-Sweden, Greenland and Svalbard. The datasets have been assembled separately and subsequently annotated for use in a comparative metagenomics analysis. Here, we present an investigation into the taxonomy and phylogenetic relationships of the functional genes recovered relating to bacterial nitrogen fixation in the four forefields. This analysis aims to contribute to the existing knowledge on pioneer microbial communities, helping to identify key genera of diazotrophic bacteria, which may have a key role building labile nitrogen stocks and soil development in oligotrophic forefield soils.

## MATERIALS AND METHODS

### Field sampling

Four Arctic glacier forefields were selected for sampling and analysis, in front of Rabots glacier (Rb), N-Sweden (67° 54′ 25.6284″ N, 18° 26′ 51.0792″ E); Storglaciaren (St), N-Sweden (67° 52′ 21.1116″ N, 18° 34′ 2.676″ E); Midtre Lovenbreen (Ml), Svalbard (79° 6′ 1.8″ N, 12° 9′ 21.996″ E) and Russell Glacier (Rl), Greenland (67° 9′ 23.4324″ N, 50° 3′ 50.342″ W). Samples were obtained in July 2013 (Midtre Lovenbreen) and July 2014 (Russell, Rabots and Storglaciaren). Surface soil from each site was sampled using a chronosequence/transect-based approach, constructing three parallel transects along the forefield moving away from the terminus (Bradley, Singarayer and Anesio 2014). Chronosequence-based sampling was used to capture the diversity in nutrient concentration and microbial taxonomy of each forefield, to make more holistic comparisons between glacial forefields. Bulk surface samples were collected into sterile Whirlpak bags, and frozen at −20°C. Observationally, the sites comprised soils at very different development stages. A ‘typical’ smooth successional chronosequence from bare ground, to more developed, plant colonised soil was observed in the Ml forefield. However, the other sites sampled had a more heterogeneous chronosequence, with earlier and often more patchy plant colonisation.

### Soil organic carbon and total nitrogen content

Soil total nitrogen (TN) and total organic carbon (TOC) were determined using mass spectrometry on a FlastEA 1112 nitrogen and carbon elemental analyser. The protocol described in Hedges and Stern (1984) was used for sample preparation. In brief, for TN analysis soil samples were weighed and dried at 50°C overnight, before subsamples were transferred into tin capsules. For TOC analysis, 2 ml of 1 M HCL was incrementally added to 0.1 g of sample (Wo) until effervescence stopped. Subsequently samples were again dried overnight at 50°C, left to equilibrate with hydroscopic salts, and re-weighed (Wf). Finally, subsamples were transferred into tin vials for analysis. The percentage of TOC in each sample was calculated using a correction for acidification induced weight change (Equation 1, Supporting Information). Where possible, three environmental replicates were analysed for each TN and TOC per sampling site.

### DNA extraction, library preparation and sequencing

As this study was focused on the microbial diversity in bulk surface soil, DNA was extracted using a Mo-Bio DNAEasy PowerSoil DNA extraction kit (QIAGEN, UK), with DNA yield quantified using a Qubit 2.0 fluorometer. Samples that yielded less than 50 ng of DNA during extractions were pooled with their field replicates prior to sequencing. This method has been previously shown to obtain high DNA yields from soils and has been used for soil microbial diversity analysis in a number of studies, including root microbiomes (Fierer *et al.*^2007^; Allison *et al.*^2008^; İnceoǧlu *et al.*^2010^; Carvalhais *et al.*^2013^; Vishnivetskaya *et al.*^2014^). However, as this approach is not directly targeting the soil rhizosphere communities, there may be limitations to DNA extraction from this subset of the microbial community. Metagenomes were sequenced using an Illumina Next-Seq 500 (Rb, St and Rl) and an Illumina-Mi Seq (Ml), with a TruSeq library prep kit at the University of Bristol Genomics facility. A total of 70 metagenomes were sequenced across the four sites using 2x 150bp (Rb, ST, Rl) and 2x 100bp (Ml) paired-end reads (Table S1, Supporting Information). Sequencing read output for each site can be identified in Table S2 (Supporting Information), ranging between 3 817 852 and 10 510 0186 reads per metagenome.

### Metagenome assembly and annotation

The 70 sequenced datasets were quality trimmed and subsequently assembled individually using the SPADES 3.10.0-dev assembler (Bankevich *et al.*^2012^). These assemblies were carried out in collaboration with the DOE Joint Genome Institute (Walnut Creek, CA), using the BFC algorithm for read error correction (Li 2015), and the --meta and --only-assembler flags. Furthermore, incremental Kmer lengths were used (22, 33, 55 and 77) to identify the most appropriate value for assembly. Assembly size for each metagenome ranged between 241660 and 429543524 bases (Table S2, Supporting Information). Functional annotation of the 70 metagenomes was subsequently carried out using the Integrated Microbial Genomes with Microbiome Samples (IMG/M) system (Chen *et al.*^2017^). Rarefaction curves were created in MG-RAST 4.0.3 for each metagenome (Figs S1–S4, Supporting Information; Meyer *et al.*^2008^). Each metagenome was evaluated based on the number of contigs assembled and species obtained, to highlight metagenomes that may be under sampled through sequencing. Under sampling can occur in highly diverse metagenomes, where the sequencing is not adequate to reveal all taxa present in the sample (Torsvik, Øvreås and Thingstad 2002). Consequently, in under sampled datasets, some organisms, particularly those which are less abundant, may not be included in the output metagenome (Rodriguez and Konstantinidis 2014).

For each metagenome, the nifH gene for nitrogen fixation was searched using the Basic Local Alignment Search Tool for Proteins (BLAST-p) with an e-value of 1e^−5^ and extracted. As nifH genes are generally found in a phylogenetically conserved nitrogenase cluster (with nif D, K, N and E), these genes were also searched for and extracted (Howard and Rees 1996). Nif genes were dereplicated, removing duplicate copies, using vsearch 2.6.0, leaving a total of 185 assembled nif genes for subsequent analysis (Rognes *et al.*^2016^). The nif genes used for the analysis have been deposited in GenBank, under accession numbers MH551286 - MH551470. Gene abundance was calculated as a combined value of nifHDKNE, normalised in relation to the abundance of the bacterial single copy housekeeping gene, rpoB, for each site (Vos *et al.*^2012^; Ishii *et al.*^2015^). As this method relies on sequencing unamplified genes, the nif gene counts are limited and may not be exhaustive for individual samples. This is particularly the case for unamplified sequencing of complex microbiome datasets, such as soil samples (Rodriguez and Konstantinidis 2014). Additionally, diazotrophs can contain multiple different nif genes, and several copies of a single variant, so should not be used as a measure to enumerate the explicit number of diazotrophs in each sample (Zehr *et al.*^2003^). Finally, the raw sequencing reads were mapped to the extracted nif contigs for each metagenome using the BWA-MEM algorithm (Li and Durbin 2009). The alignment score (AS) of each read/contig is reported, which numerically indicates the quality of the alignments.

### Nif taxonomy

The taxonomic distribution of all nif sequences (HDKNE) was carried out using a Last Common Ancestor (LCA) analysis in MEGAN 6.9.0 (Huson *et al.*^2016^). For each forefield, nifHDKNE sequences were nucleotide BLAST (BLASTn) searched against an NCBI GenBank database of complete bacterial genomes. The sequences were subsequently binned based on the NCBI taxonomy, using an LCA algorithm, and visualised at the genus level for each forefield (Huson *et al.*^2016^).

### Gene phylogeny

A phylogeny for nifH, based on clusters identified in Zehr *et al*. (2003), was carried out, as this gene is supported by the largest body of research. Sample nifH sequences were aligned to sequences of cultured isolates, largely derived from the phylogeny by Deslippe and Egger (2006). GenBank and UniProtKB accession numbers for cultured isolates are available in Table S3 (Supporting Information). DNA sequence alignments were generated in SATé 2.2.7, using MAFT, MUSCLE and FASTTREE (Liu *et al.*^2011^). The GTR+CAT model was implemented, with the decomposition set to longest (to minimise long branch attraction) and a maximum number of iterations set to 8. Alignments were manually edited in Mesquite, alongside generating Nexus and Phylip format files (Maddison and Maddison 2017). Maximum likelihood phylogenies were carried out using the CIPRES implementation of RAXML-HPC2 8.2.10 on XSEDE^1^, with 1000 bootstrap iterations (Stamatakis 2014). The GTR+G model of nucleotide substitutions was implemented, as identified with j model test (Guindon and Gascuel 2003; Darriba *et al.*^2012^). Trees were evaluated using Figtree 1.4.3^2^, before annotation with EvolView v2^3^ (He *et al.*^2016^). Graphical enhancements were made using Inkscape 0.92.2^4^. Comparisons between nifH sample sequences and cultured isolates were made using NCBI BLASTn^5^, to identify nearest cultured relatives.

## RESULTS AND DISCUSSION

### Soil carbon and nitrogen

The range of values obtained within and between forefields for TOC and TN for samples from each forefield is listed in Table 1. These values include TOC and TN from both microbial and plant sources. Looking at average nutrient contents, comparing across the forefields, TN content ranges from averages below detection to 1.95 mg g^−1^, between St and Rl, respectively (Table 1). TOC content follows the same trend, increasing from the two Swedish glaciers (St and Rb), to Ml and Rl. Results from a one-way ANOVA analysis for each nutrient did not show any statistically significant differences in the TN measured between forefields (*P* > 0.05). However, concentrations of TOC were found to vary significantly (*P* = 0.002) (Table S4, Supporting Information). Additional analysis of the TOC variance between forefields using a post-hoc Tukey analysis revealed the significant difference was between the St and Rl forefields, with Rl containing almost 10 times the TOC content of St on average (*P* < 0.01, Table 1; Table S5, Supporting Information).

**Table 1.** Summary statistics for TN and TOC across the four forefields (Midtre Lovenbreen Ml, Russell Rl, Storglaciaren St and Rabots Rb). The average, minimum, maximum and standard deviation (SD) across each forefield is given. The detection limit for both TN and TOC was 0.1 mg g^−1^. Sites recording values below detection (b.d) are shown.

TN (mg g^−1^)	Average	Minimum	Maximum	SD
Ml	b.d.	b.d	4.90	1.56
Rl	1.95	b.d	6.94	2.15
St	b.d.	b.d	4.19	0.93
Rb	1.04	b.d	3.35	1.33
TOC (mg g^−1^)	Average	Minimum	Maximum	SD
Ml	10.56	b.d.	72.36	21.14
Rl	26.36	b.d.	82.70	26.35
St	2.78	b.d.	27.89	6.25
Rb	6.81	b.d.	22.90	9.66

Samples from the Rl forefield revealed the widest range in both TOC (below detection—82.70mg g^−1^) and TN (below detection—6.94mg g^−1^), respectively (Table 1). This contrasts with the Rb forefield, where TOC and TN values expressed a smaller range, from below detection to 22.90 mg g^−1^ and below detection up to 3.35 mg g^−1^, respectively. A range of values is expected across sites within each forefield, due to soil development which takes place over successional chronosequences and given variations in sources of autochthonous and allochthonous material (Bradley, Singarayer and Anesio 2014), for example, in the deposition of aeolian material (such as soot), or the presence of ancient *in situ* organic pools, exposed by glacier retreat (Tranter *et al*. 2005; Schulz *et al.*^2013^; Bradley *et al.*^2015^). For example, across the Ml chronosequence TN and TOC increase from below detection and 2.85 mg g^−1^, to 4.4 mg g^−1^ and 14.5 mg g^−1^, in line with expected soil development (Table S6, Supporting Information; Bradley *et al.*^2016^). However, whilst differences in soil nutrient content do occur between sites, the values fall into the general range observed from other forefields (1–2 mg g^−1^ nitrogen, and 0.1–40 mg g^−1^ carbon) (Bradley, Singarayer and Anesio 2014) and are indicative of a generally oligotrophic environment.

### Rarefaction analysis

Rarefaction analysis was used to investigate the coverage of diversity in each metagenome, identifying any datasets where species content may be under sampled (Figs S1–S4, Supporting Information). For each forefield, an assortment of both adequately sequenced and under sampled metagenomes was obtained (Figs S1–S4, Supporting Information). Metagenomes that show rarefaction curves to reach saturation are likely to adequately profile the microbial diversity in the samples, for example metagenomes Ml 7, Rl 15, St 16 and St 17 (Figs S1, S2 and S3, Supporting Information). However, those metagenomes in which species number does not reach saturation are most likely to exclude taxa, for example ML1, ML 20, Rl 14 and Rl 20 (Fig. S1 and S2, Supporting Information). In these metagenomes, the least abundant taxa are most probably excluded from the dataset, due to the reduced abundance of DNA for sequencing from these organisms (Rodriguez and Konstantinidis 2014). Whilst this does not detract from conclusions drawn on the organisms present in the samples, the full depth of diversity in under sampled metagenomes cannot be highlighted. This issue is often prevalent in highly complex datasets such as soil and can only be resolved through continued deeper sequencing of those metagenomes (Rodriguez and Konstantinidis 2014).

### Nif genes recovered

**Figure 1.**
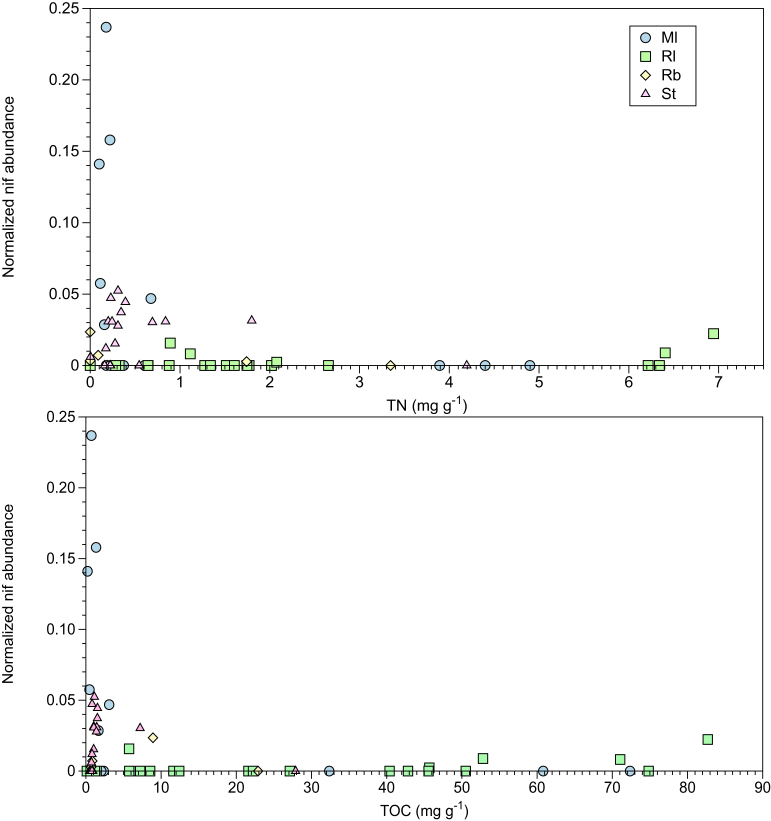
Relationship between normalised nif gene abundance (nifHDKNE) and concentration of TOC and TN per gram of soil, across all sampling sites. Nif gene abundance values are normalised against the bacterial single copy housekeeping gene, rpoB, for each metagenome. Values across the different forefields are noted, including: Midtre Lovenbreen (Ml), Russel (Rl), Rabots (Rb) and Storglaciaren (St).

The total abundance of dereplicated *rpoB* normalised contigs containing nif genes (nifHDKNE), in relation to the variation of TN and TOC, spanning all sampling sites is shown in Fig. 1. A total of 185 nif genes contained on assembled contigs were recovered from the datasets. In 75% of samples where nif genes were detected, the TN and TOC concentrations fell below 1 and 5 mg g^−1^, respectively (Fig. 1). Conversely, in samples where nif genes were not detected, 61% and 49% measured below 1 and 5 mg g^−1^, of TN and TOC, respectively (Fig. 1). As sequencing output varied substantially between metagenomes, further sequencing may reveal additional genes due to the complex nature of soil microbiome samples (Table S2, Supporting Information; Rodriguez and Konstantinos 2014). However, this may indicate that samples with limited TN/TOC could have a larger relative abundance of genes for diazotrophy, as these were recovered through the sequencing effort undertaken. Interestingly, a similar trend between nitrogen fixation and TN has been reflected by the assays carried out by Telling *et al*. (2011), whereby fixation rates on Arctic glaciers were negatively correlated with total inorganic nitrogen content. Additionally, a link between nif gene abundance and activity is supported theoretically, as fixation becomes less metabolically beneficial when labile nitrogen stocks increase (Gutschink *et al.*^1978^). When applied to forefield soils, both TN and TOC have been shown to increase over successional chronosequences, indicating nitrogen fixation may become less profitable with soil development (Duc *et al.*2009a; Brankatschk *et al.*^2011^; Bradley, Singarayer and Anesio 2014). Furthermore, research by Brankatschk *et al*. (2011) identified a link between nif gene abundance and enzyme activity, indicating sites with high numbers of nif genes, such as Storglaciaren, would have enhanced nitrogen fixation activities. However, the relationship between gene abundance and nitrogen fixation activity is not always fully defined, as areas with low nitrogenase activity have previously been linked to high gene abundance in the Damma Glacier (Swiss Alps) (Duc *et al.*2009a).

The results of mapping sequencing reads to the nif genes is provided in Table S7 (Supporting Information). This highlights the Alignment Score (AS), which indicates the alignment quality between reads and contigs (Table S7, Spporting Information). The number of nif genes for each score threshold is provided, alongside the percentage of reads with AS over 60. The Alignment Score ranges between 0 and the maximum length of the reads (0–100 for MI dataset and 0–150 for Rb, St and RI datasets). For each forefield, the percentage of alignments with an AS greater than 60 was 1.06x10^−3^ (Ml), 4.23x10^−5^ (Rl), 2.38x10^−4^ (Rb) and 9.56x10^−4^ (St). Plots of the normalised nif genes recovered and the number of reads aligning to genes with an AS over 60, for each metagenome, are available in Figs S5–S8 (Supporting Information).

### Nitrogenase clusters

**Figure 2.**
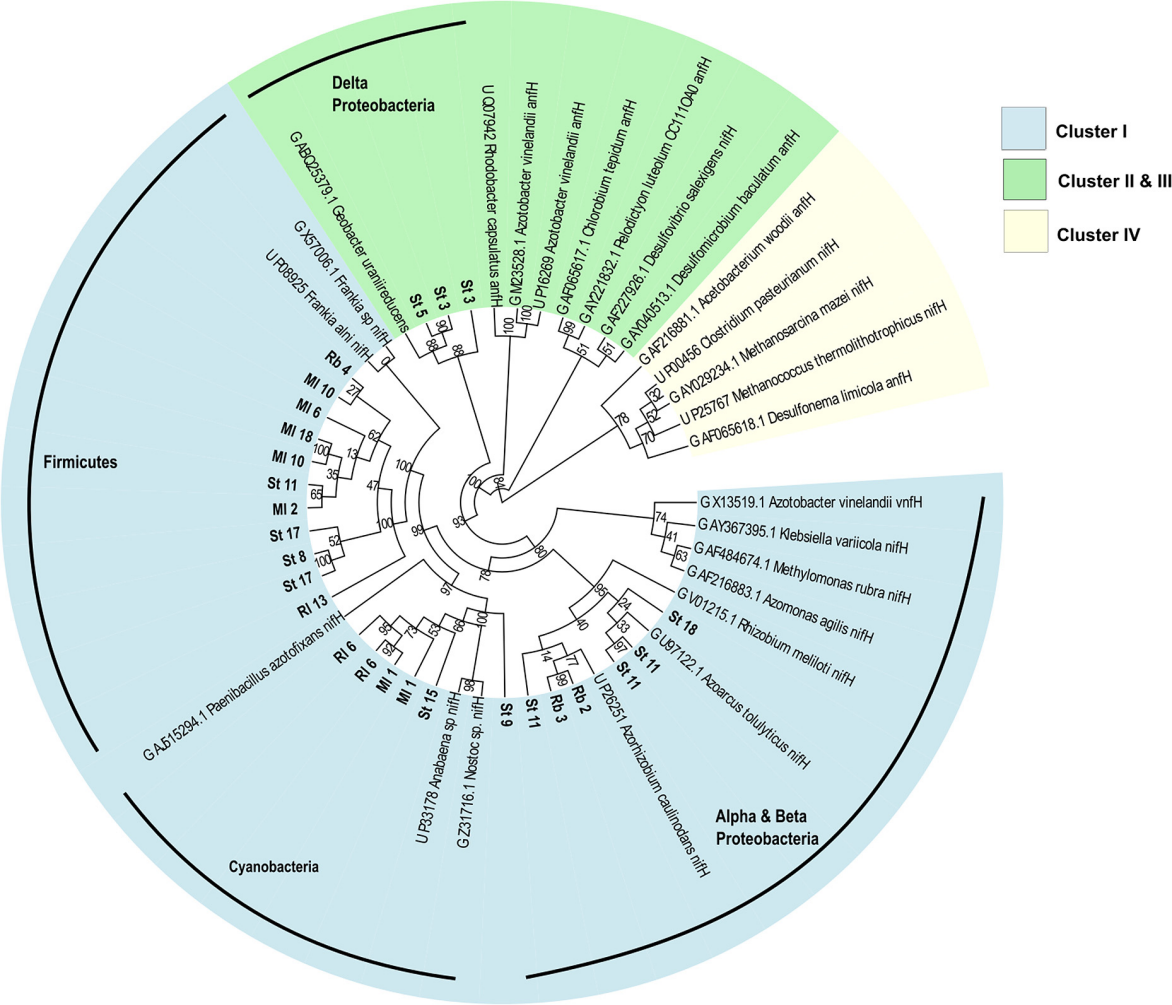
nifH maximum likelihood phylogeny of sample sequences (bold) and sequenced samples derived from NCBI GenBank and UniProtKB. Most sample sequences were obtained from the nifH phylogeny of Deslippe and Egger (2006). For study samples, the Sample ID is given, corresponding to Table S1 (Supporting Information). For sequenced samples, the database, organism name and gene are given. Bootstrap support values are given, based on 1000 tree iterations. The nifH clusters (derived from Zehr *et al.*^2003^) are denoted by leaf colours (Cluster I-IV). The tree is rooted on Cluster IV, as this group contains divergent ‘nif-like’ sequences (Zehr *et al.*^2003^). Key groups containing sample sequences are noted, including Firmicutes, Cyanobacteria, Alphaproteobacteria, Betaproteobacteria and Deltaproteobacteria.

Our newly sampled bacteria were analysed and grouped with previously published relatives, as shown in Zehr *et al*. (2003). Forefield sequences were distributed across Cluster I (23 sample sequences) and III (3 sample sequences), with no representatives in Cluster II or IV (Fig. 2). Thus, 88.5% of sample sequences were attributed to Cluster I, which contains the typical Mo nifH, indicating the presence of plentiful molybdenum in soils for the nitrogenase cofactor (Zehr *et al.*^2003^).

Environmental samples in Cluster I included the groups Alphaproteobacteria, Betaproteobacteria, Cyanobacteria and Firmicutes (Fig. 2). The first group, associated with Alphaproteobacteria and Betaproteobacteria, incorporated five environmental samples that clustered most closely with *Azorhizobium caulinodans* and *Azoarcus tolulyticus*. These are plant-associated diazotrophs, important for establishing stocks of fixed nitrogen for legume uptake, supporting plant growth (Hurek and Hurek 1995; Dreyfus, Garcia and Gillis 1988). The second group was comprised of six sample sequences, clustering with the Cyanobacteria, *Nostoc* and *Anabaena*, which are free living nitrogen fixers (Zehr *et al.*^2003^). Cyanobacteria have been proposed as crucial for building labile nitrogen pools in newly exposed soils, important for facilitating heterotroph colonisation, and have been identified in other forefields using SSU rRNA amplicon sequencing (Schmidt *et al.*^2008^; Duc *et al.*2009a; Frey *et al.*^2013^). Group 3 contained 11 highly related sample nifH sequences, grouping closely to *Frankia*. This genus is composed of nitrogen fixing bacteria that are symbionts of actinorhizal plant roots, and again provides evidence for bacterial support of plant growth and establishment, through supplies of fixed nitrogen (Benson and Silvester 1993). Whilst the forefields may have a low diversity of root symbiotic diazotrophs, this may also relate to sub-optimal cell lysis and separation of root-associated cells during the DNA extraction process, or that these organisms were at a low abundance and thus not captured through sequencing.

Environmental samples were also present in Cluster III, which is attributed to a group of anaerobic bacteria (Zehr *et al.*^2003^). The three sample sequences clustered most closely to *Geobacter uraniireducens*, an anaerobe common in sediments under metal reducing conditions, capable of dissimilatory Fe(III) reduction (Shelobolina *et al.*^2008^). However, no sample sequences were linked to Cluster II, which is associated with organisms containing the alternative anfH, containing an Fe–Fe cofactor, used in the absence of molybdenum and Vanadium (Zehr *et al.*^2003^).

These results reflect those of Duc *et al*. (2009a), who used clone libraries to evaluate the phylogeny of diazotrophs across the Damma Glacier, Switzerland. Interestingly, nifH sequences from their analysis also grouped with nitrogenase Clusters I and III (Duc *et al.*2009a). Additionally, genera identified by Duc *et al*. (2009a) included the key genera identified in this analysis, such as *Geobacter, Nostoc* and *Anabaena*, suggesting that these organisms are common across forefields (Duc *et al.*2009a). The prevalence of these organisms may be due to adaptations or attributes to cold environments, such as cold or UV tolerance, and the release of protective exudates (Tamaru *et al.*^2005^; Chattopadhyay 2006; Pattanaik, Schumann and Karsten 2007). Cyanobacteria such as *Nostoc* have been shown to produce extracellular polysaccharides (EPS) which are important for desiccation and freeze-thaw tolerance in Arctic environments (Tamaru *et al.*^2005^). *Geobacter* are commonly found in anaerobic environments, and therefore may tolerate any anoxia in forefield soils created by frequent meltwater flooding and the formation of melt pools (Duc *et al.*2009a). The consistent identification of *Geobacter*, *Nostoc* and *Frankia* in forefield soils using nifH analysis indicates that a core group of diazotrophs may be present across Arctic forefields. These diazotrophs may be important for facilitating plant colonisation and establishment, either by building labile pools in newly exposed soils (Cyanobacteria) or through symbiosis (*Frankia, Azorhizobium*).

Results from BLASTn searching each nifH sequence against cultured isolates revealed forefield sample sequences were divergent, with sequence identity ranging between 80%–95% (Table S8, Supporting Information). This indicates that the diazotrophs present in the samples are novel compared to those which have been previously identified and may be unique or contain adaptations to cold oligotrophic forefield conditions. However, as less abundant organisms will contribute to a minor proportion of the unamplified sequenced DNA and nifH gene pool, using additional nif genes may help highlight the presence of rare organisms in samples (Cowan *et al.*^2005^). This may be especially helpful for metagenomes where sequencing coverage was not sufficient to profile the complete community structure, and thereby some low abundance organisms may not have been represented in the final dataset (Figs S1–S4, Supporting Information).

### Diazotroph community structure

**Figure 3.**
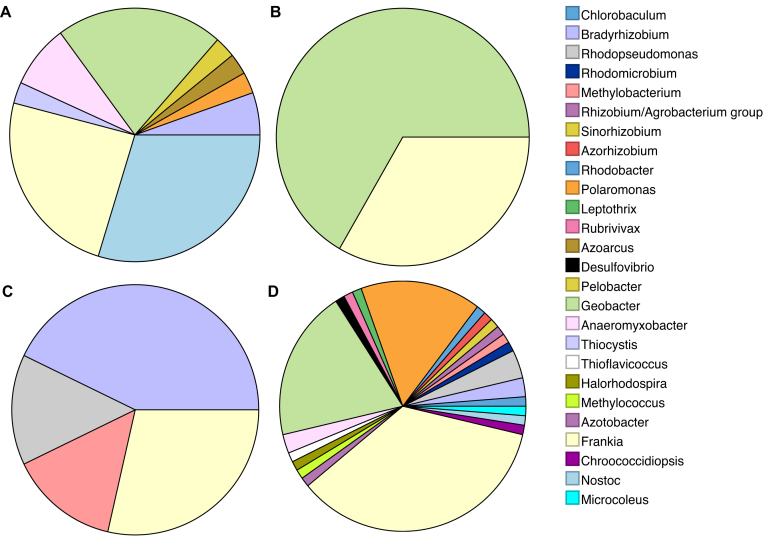
Taxonomic distribution of nif (HDKNE) genes for each forefield at the genus level: Midtre Lovenbreen Ml (**A**), Russell Rl (**B**), Rabots Rb (**C**), Storglaciaren St (**D**). The total nif gene sequence count for each site was 42, 15, 13 and 91, respectively.

LCA analysis with multiple nif genes (HDKNE) identified the key organisms consistent between two or more forefields, including *Geobacter*, *Frankia* and *Nostoc*, which were also highlighted in the nifH analysis. Additional genera, for example *Polaromonas, Pelobacter and Microcoleus* were also identified here through the inclusion of additional nif genes (nifDKNE) (Fig. 3). This suggests including multiple nitrogenase genes provides a more holistic view of the diazotroph community structure in each forefield, due to the low copy number of these genes in unamplified samples. This is a particular issue of highly diverse metagenome samples, such as those from soils, as sequencing depth may not profile the complete community structure (Rodriguez and Konstantinidis 2014).

The assignment of nif genes in the Rl forefield covers two key genera, *Geobacter* and *Frankia*. Limited research has been conducted into the presence of *Frankia* in Greenland; however, these organisms are typically associated with common actinorhizal plants (Benson and Silvester 1993; Chaia, Wall and Huss-Danell 2010). This group forms nitrogen fixing root nodules with *Frankia* in exchange for reduced carbon and therefore are commonly found as early colonisers of undeveloped, oligotrophic soils (Wall 2000; Schwinter 2012). This is in agreement with the limited nitrogen content detected in this forefield, at 2.04 TN g^−1^ (Fig. 1 and Table 1). Additionally, the presence of plants has been identified as a key control on microbial community structure over the Damma Glacier forefield, Switzerland (Miniaci *et al.*^2007^). Furthermore, the identification of the anaerobic *Geobacter* indicates the presence of periodically saturated and anoxic conditions along the forefield, possibly attributed to meltwater flooding (Duc *et al.*2009a). *Geobacter* are dissimilatory metal and sulphur reducing bacteria and have been proposed as key players in sediment nutrient cycles, oxidation of organic matter, bioremediation and soil gleying (Lovley 1993; Childers *et al.*^2002^; Methe *et al.*^2003^). *Geobacter* have been consistently identified across glacier forefield soils, which may relate to their metabolic diversity, thereby making these organisms well suited to fluctuating environmental conditions in forefield soils (Duc *et al.*2009a,b; Edwards and Cook 2015; Rime, Hartmann and Brunner 2015). This group has been shown to use chemotaxis to access Fe(III) oxides as an electron acceptor, which may explain their prevalence over other non-motile Fe(III) reducers (Rime, Hartmann and Brunner 2015). Whilst deeper sequencing in some metagenomes may highlight additional rare diazotrophic bacteria in Rl samples, it is likely that *Geobacter* and *Frankia* were the most dominant nitrogen fixers present, as these were identified through direct sequencing of unamplified DNA (Cowan *et al.*^2005^; Fig. S2, Supporting Information).

Similarly to Rl, the taxonomic diversity detected in the N-Swedish Rb forefield was largely comprised of root associated diazotrophs, including the genera *Bradyrhizobium*, *Frankia, Methylobacterium* and *Rhodopseudomonas* (Fig. 3). This may relate to the lack of bare soil observed at this forefield, and therefore limited requirement for free living diazotrophs (Miniaci *et al.*^2007^). This site also had a low average soil nitrogen content, at 1.04 mg g^−1^ (Fig. 1 and Table 1), which, alongside the detection of *Rhizobia*, Fabaceae root-nodule symbionts, indicates that nitrogen limitation for plant growth may have been occurring in soils (Mylona, Pawlowski and Bisseling 1995). Actinorhizal and legume plants, which directly benefit from biological nitrogen fixation through symbiosis, such as Clover, are likely to prevail in developing forefield soils (Fagerli and Svenning 2005; Chaia, Wall and Huss-Danell 2010). This is because they maintain a competitive advantage over other plants in nitrogen limited conditions, typical of newly exposed soils (Menge and Hedin 2009; Bradley, Singarayer and Anesio 2014). Additionally, Rb had a lower average soil TOC content than other forefields, at 6.8 mg g^−1^ (Fig. 1 and Table 1). Thus, *Rhizobia* are likely to benefit from symbiosis with plants through the supply of reduced carbon (Denison and Kiers 2004). Plants may therefore be acting as a control on the forefield microbial community structure, endorsing the presence of root-associated diazotrophs (Miniaci *et al.*^2007^). Rarefaction curves for Rb sites were shown to be nearing saturation, indicating much of the microbial community structure was profiled (Fig. S4, Supporting Information). Additional sequencing for these samples may reveal further low abundance taxa; however, it is likely that the most dominant fraction of diazotrophs have been identified adequately through our analysis.

The nif genes recovered from the Ml forefield showed a wider taxonomic diversity of diazotrophs and contained sequences linked to the genera *Nostoc, Polaromonas*, *Bradyrhizobium*, *Pelobacter*, *Azoarcus* and *Anaeromyxobacter*. The presence of the Cyanobacteria, *Nostoc*, was expected due to the greater extent of bare soil observed in this forefield, enhancing the need for early colonisers (Frey *et al.*^2013^). Additionally, EPS production enables this group to resist harsh freeze-thaw cycles, common in Arctic environments (Tamaru *et al.*^2005^). Given the high latitude of this forefield, it is also not surprising to find *Polaromonas*, which are known psychrophiles (Irgens, Gosink and Staley 1996). The presence of *Bradyrhizobium* and *Frankia* indicate plants may require additional fixed nitrogen through symbiosis, corresponding with the low nitrogen stocks detected (Benson and Silvester 1993; Mylona, Pawlowski and Bisseling 1995; Chaia, Wall and Huss-Danell 2010; Fig. 1 and Table 1). Additionally, the presence of legume symbiotic diazotrophs is interesting, as Fabaceae are non-native to Svalbard, having been introduced over the 20th Century (Fagerli and Svenning 2005). The absence of early plant colonisation in the forefield may also have been a control on overall microbial community structure, endorsing a range of non-symbiotic diazotrophs (Knelman *et al.*^2012^). Alongside *Geobacter*, the identification of *Pelobacter, Thiocystis* and *Anaeromyxobacter*, again indicates permanent or periodic anaerobic conditions in the glacier forefield, similarly to Rl (Schink and Stieb 1983; Sanford *et al.*^2002^). *Pelobacter* are anaerobic organisms containing diverse fermentative metabolisms, which may make this group well suited to the rapidly changing conditions in forefield soils (Schink 2006). For example, *Pelobacter* have been shown to ferment acetylene using acetylene hydratase to acetate for cell growth or using nitrogenase to ethylene through nitrogen fixation (Akob *et al.*^2017^). The genomic results for the Ml forefield falls in line with 16s amplicon data presented by Bradley *et al*. (2016). This study also found *Frankia, Rhizobium, Nostoc* and *Geobacter* in the Ml forefield (Bradley *et al.*^2016^). The identification of additional organisms such as *Devosia*, *Sphingomonas* and *Rhodoplanes* may relate to the use of amplification in their methodology, thereby aiding the discovery of low abundance organisms (Bradley *et al.*^2016^). Additionally, some metagenomes from this forefield would have benefitted from greater sequencing depth in order to completely profile the microbial community composition (Fig. S1, Supporting Information). Therefore, deep sequencing of these samples may reveal additional low abundance diazotrophs, unidentified in this analysis.

Finally, the St forefield contained sequences relating to *Nostoc, Geobacter, Rhizobium, Polaromonas* and *Frankia*, in line with the other forefields sampled (Fig. 3). This supports the identification of a core group of diazotrophs present across Arctic glacier forefields. However, several diazotrophs detected at this site may also have importance in sulphur cycling, alongside nitrogen fixation (Fig. 3). The detection of the anaerobic diazotrophs *Geobacter* and *Desulfovibrio* indicates the potential for sulphur reduction, whereby energy is gained through reducing sulphur (S) or sulphate (SO_4_^2−^) to hydrogen sulphide (H_2_S), with the oxidation of organic carbon (Boopathy and Kulpa 1993; Caccavo *et al.*^1994^). However, inorganic S and SO_4_ have been found to be limiting for both plants and microbes in newly exposed glacier forefield soils (Allison *et al.*^2007^; Prietzel *et al.*^2013^). Nevertheless, desulphonating bacteria, whom metabolise organically bound sulphur to labile sulphates, have been found in forefield soils, and may therefore help overcome S limitation (Schmalenberger and Noll 2009; Prietzel *et al.*^2013^). Additionally, suitable anaerobic growth conditions for sulphur reducing bacteria may occur frequently in stagnated proglacial meltwater pools and during periods of meltwater flushing (Duc *et al.*2009a). Furthermore, the detection of organisms such as *Chlorobaculum, Thioflavicoccus, Halorhodospira and Thiocystis* indicates the potential for St forefield bacteria to carry out both nitrogen fixation and sulphur oxidation (Fig. 3). These organisms have the potential to oxidise H_2_S to S and SO_4_, alongside gaining fixed nitrogen through diazotrophy (Imhoff and Pfenning 2001; Chan, Morgan-Kiss and Hanson 2008; Peduzzi *et al.*^2011^; Challacombe *et al.*^2013^). The ability of these organisms to overcome nitrogen limitation through fixation, and to respire anaerobically in anoxic soils, may make this group well suited to harsh forefield environments. Additionally, as *Halorhodospira* is also halophilic, this may indicate resistance to high salinity environments, such as ice brine channels, or evaporation ponds in the St forefield (DasSarma and DasSarma 2006).

The diazotroph community composition observed using LCA nifHDKNE analysis was again largely consistent with those found at the Damma Glacier, Switzerland (Duc *et al.*2009a,b; Frey *et al.*^2013^). This includes genera such as *Methylobacterium*, *Bradyrhizobium*, *Azotobacter*, *Anabaena*, *Nostoc* and *Geobacter* (Duc *et al.*2009a). This supports the results from the nifH phylogeny, indicating the presence of consistent genera across forefields, which may be well adapted to the cold, oligotrophic and high UV conditions. Plant colonisation has also been identified as an influence on the diazotrophic community composition, in agreement with studies on the Damma Glacier, Switzerland (Miniaci *et al.*^2007^; Duc *et al.*2009a; Zumsteg *et al.*^2012^). However, it is important to acknowledge that additional factors, such as latitude, bedrock minerology, organic matter and aeolian nitrogen deposition, may also have an influence on diazotroph community structure and abundance (Duc *et al.*2009a; Zumsteg *et al.*^2012^). Some genera found by Duc *et al*. (2009a), such as *Oscillatoria*, *Ideonella* and *Paenibacillus* were not identified in this study (Fig. 3). This may relate to the absence of these organisms in the four forefields in this analysis, but also may relate to the alternate approach used. As this analysis uses unamplified nifH sequences, some low abundance organisms may not be sequenced due to incomplete sequencing depth in highly complex samples (Rodriguez and Konstantinos 2014; Figs S1–S4, Supporting Information). Thus, it cannot be ruled out that these organisms were also not present in the forefields, but at a lower abundance than those captured by the sequencing effort (Prakash and Taylor 2012). In order to profile the complete community of some metagenomes, including low abundance organisms, deeper sequencing would be required, due to the diverse nature of soil samples (Rodriguez and Konstantinos 2014). Despite this, this analysis has been able to capture a diverse group of diazotrophs that appear to be common across glacier forefields and are likely the most abundant fraction of the nitrogen fixing community, as these were captured by unamplified DNA sequencing (Rodriguez and Konstantinos 2014.

Overall, this study has used a nifH phylogeny to identify a key group of diazotrophs across four Arctic forefields, associated with both Cluster I and III nitrogenase, linked to aerobic and anaerobic organisms containing the typical Mo nifH (Zehr *et al.*^2003^). Incorporating multiple nif genes (HDKNE) revealed additional organisms from unamplified metagenome samples, compared to using the nifH gene exclusively. This may relate to the complex nature of soil metagenome samples, whereby sequencing depth is not always adequate to profile the complete microbial community diversity. Thus, to reveal all low abundance diazotrophs, some metagenomes would require additional deep sequencing. Key diazotrophs were found to be metabolically diverse, including genera such as *Geobacter*, *Frankia*, *Nostoc, Polaromonas* and *Bradyrhizobium*. A range of diazotrohic organisms outside the key group were also highlighted, including halophiles, psychrophiles and bacteria associated with fermentative metabolisms and sulphur cycling. Therefore, this analysis has shown a diverse group of diazotrohic bacteria present in Arctic forefield soils, including a consistent core subset. These diazotrophs have the potential to build labile nitrogen stocks in forefield soils, which may support further colonisation and soil development.

## Supplementary Material

Supplementary Data

## Footnotes

1
https://www.phylo.org/

2
http://tree.bio.ed.ac.uk/software/figtree/

3
http://www.evolgenius.info/evolview/

4
https://inkscape.org/en/

5
https://blast.ncbi.nlm.nih.gov/
