# Supplementary material for: Metagenomic insights into diazotrophic communities across Arctic glacier forefields
Source: FEMS Microbiol Ecol. 2018 Jun 12;94(9):fiy114. doi: 10.1093/femsec/fiy114 (PMC6054269; doi:10.1093/femsec/fiy114)
Supplement: Supplementary Data [file fiy114_supplemental_files.docx]

**Supplementary Information**

***Equation S1:*** *Correction for weight change during acidification of samples for organic carbon elemental analysis.*

$$\%OC=\left[ \frac{100 x mgOC}{mg sample} \right]x \left[ \frac{Wf}{\mathrm{Wo}} \right]$$

Where Wo is sediment dry weight prior to acidification, and Wf is dry weight after acidification.

***Table S1:*** *Samples used in metagenomic sequencing across the four Arctic forefields, and the corresponding metadata. Samples were obtained in a transect across each forefield, moving away from the glacier terminus. For the Midtre Lovenbreen (Ml), Russell (Rl) and Storglaciaren (St) sites, three parallel transects were conducted to obtain field replicates. This was not possible for the Rabots (Rb) site.*

| **Site ID** | **Forefield** | **Latitude** | **Longitude** | **Altitude (m)** | **Date obtained** |
| --- | --- | --- | --- | --- | --- |
| Ml 1 | Ml | 79.100555 | 12.156111 | 54 | 29/07/2013 |
| Ml 2 | Ml | 79.112223 | 12.175555 | 44 | 29/07/2013 |
| Ml 3 | Ml | 79.112222 | 12.258333 | 44 | 29/07/2013 |
| Ml 4 | Ml | 79.118333 | 12.093611 | 54 | 29/07/2013 |
| Ml 5 | Ml | 79.113611 | 12.195833 | 52 | 29/07/2013 |
| Ml 6 | Ml | 79.104444 | 12.278888 | 52 | 29/07/2013 |
| Ml 7 | Ml | 79.152555 | 12.215555 | 50 | 29/07/2013 |
| Ml 8 | Ml | 79.151388 | 12.253611 | 43 | 29/07/2013 |
| Ml 9 | Ml | 79.140833 | 12.092222 | 43 | 29/07/2013 |
| Ml 10 | Ml | 78.927777 | 12.254166 | 35 | 29/07/2013 |
| Ml 11 | Ml | 78.921666 | 12.076666 | 40 | 29/07/2013 |
| Ml 12 | Ml | 78.907777 | 12.164444 | 48 | 29/07/2013 |
| Ml 13 | Ml | 78.900555 | 12.076111 | 30 | 29/07/2013 |
| Ml 14 | Ml | 78.900555 | 12.076111 | 40 | 29/07/2013 |
| Ml 15 | Ml | 78.900555 | 12.076111 | 105 | 29/07/2013 |
| Ml 16 | Ml | 78.99 | 12.082777 | 29 | 29/07/2013 |
| Ml 17 | Ml | 78.991666 | 12.233333 | 30 | 29/07/2013 |
| Ml 18 | Ml | 78.978888 | 12.332222 | 30 | 29/07/2013 |
| Ml 19 | Ml | 79.768333 | 12.143611 | 19 | 29/07/2013 |
| Ml 20 | Ml | 79.768333 | 12.143611 | 19 | 29/07/2013 |
| Ml 21 | Ml | 79.768333 | 12.143611 | 19 | 29/07/2013 |
| Ml 22 | Ml | 79.484166 | 12.092222 | 105 | 29/07/2013 |
| Ml 23 | Ml | 79.484166 | 12.092222 | 105 | 29/07/2013 |
|  |  |  |  |  |  |
| Rl 1 | Rl | 67.15650902 | -50.06398397 | 439.168243 | 24/07/2014 |
| Rl 2 | Rl | 67.15651598 | -50.06386997 | 440.06958 | 24/07/2014 |
| Rl 3 | Rl | 67.15655998 | -50.06389101 | 439.119476 | 24/07/2014 |
| Rl 4 | Rl | 67.16295303 | -50.01826898 | 589.524109 | 25/07/2014 |
| Rl 5 | Rl | 67.16301103 | -50.018445 | 589.374084 | 25/07/2014 |
| Rl 6 | Rl | 67.16306903 | -50.01828399 | 589 | 25/07/2014 |
| Rl 7 | Rl | 67.15211598 | -50.04869697 | 515.023315 | 26/07/2014 |
| Rl 8 | Rl | 67.15208103 | -50.04859303 | 516.628906 | 26/07/2014 |
| Rl 9 | Rl | 67.15210701 | -50.04851701 | 516.909973 | 26/07/2014 |
| Rl 10 | Rl | 67.15685402 | -50.08261903 | 404.632538 | 26/07/2014 |
| Rl 11 | Rl | 67.15680499 | -50.082499 | 403.543518 | 26/07/2014 |
| Rl 12 | Rl | 67.15684304 | -50.08236104 | 404.174896 | 26/07/2014 |
| Rl 13 | Rl | 67.15642001 | -50.08365101 | 403.374634 | 26/07/2014 |
| Rl 14 | Rl | 67.15646099 | -50.08366501 | 403.970734 | 26/07/2014 |
| Rl 15 | Rl | 67.15638698 | -50.08376702 | 403.255737 | 26/07/2014 |
| Rl 16 | Rl | 67.15559204 | -50.08486102 | 411.722473 | 26/07/2014 |
| Rl 17 | Rl | 67.15558601 | -50.08499002 | 411.14502 | 26/07/2014 |
| Rl 18 | Rl | 67.155672 | -50.08488097 | 411.708984 | 26/07/2014 |
| Rl 19 | Rl | 67.08225802 | -50.32251497 | 251.438843 | 27/07/2014 |
| Rl 20 | Rl | 67.08222499 | -50.322435 | 238.636765 | 27/07/2014 |
| Rl 21 | Rl | 67.08218602 | -50.32229704 | 237.819305 | 27/07/2014 |
| Rl 22 | Rl | 67.05702002 | -50.45979604 | 147.405701 | 27/07/2014 |
| Rl 23 | Rl | 67.057002 | -50.45969403 | 148.57132 | 27/07/2014 |
| Rl 24 | Rl | 67.05692296 | -50.45960804 | 148.330139 | 27/07/2014 |
|  |  |  |  |  |  |
| Rb 1 | Rb | 67.910855 | 18.470863 | 1250m | 07/02/2014 |
| Rb 2 | Rb | 67.907119 | 18.447522 | 1105m | 07/02/2014 |
| Rb 3 | Rb | 67.907119 | 18.447522 | 1105m | 07/02/2014 |
| Rb 4 | Rb | 67.906846 | 18.44555 | 1110m | 07/02/2014 |
| Rb 5 | Rb | 67.872223 | 16.713705 | 1054m | 07/02/2014 |
|  |  |  |  |  |  |
| St 1 | St | 67.904568 | 18.607115 | 1131m | 07/01/2014 |
| St 2 | St | 67.904687 | 18.610965 | 1103m | 07/01/2014 |
| St 3 | St | 67.904687 | 18.610965 | 1103m | 07/01/2014 |
| St 4 | St | 67.904687 | 18.610965 | 1103m | 07/02/2014 |
| St 5 | St | 67.899243 | 18.344347 | 1147m | 07/01/2014 |
| St 6 | St | 67.899244 | 18.344371 | 1147m | 07/01/2014 |
| St 7 | St | 67.900853 | 18.44175 | 1146m | 07/01/2014 |
| St 8 | St | 67.900853 | 18.44175 | 1146m | 07/01/2014 |
| St 9 | St | 67.900853 | 18.44175 | 1146m | 07/01/2014 |
| St 10 | St | 67.900879 | 18.43474 | 1130m | 07/01/2014 |
| St 11 | St | 67.900879 | 18.43474 | 1130m | 07/01/2014 |
| St 12 | St | 67.900879 | 18.43474 | 1130m | 07/01/2014 |
| St 13 | St | 67.901082 | 18.428257 | 1113m | 07/01/2014 |
| St 14 | St | 67.901082 | 18.428257 | 1113m | 07/01/2014 |
| St 15 | St | 67.865505 | 16.714941 | 1103m | 07/01/2014 |
| St 16 | St | 67.865505 | 16.714941 | 1103m | 07/01/2014 |
| St 17 | St | 67.865505 | 16.714941 | 1103m | 07/01/2014 |
| St 18 | St | 67.903128 | 18.604355 | 1182m | 07/01/2014 |

***Table S2:*** *Output statistics for metagenome sequencing and assembly, for each site. The number of raw reads returned from sequencing is given, alongside the subsequent assembly sizes, in both sequences and bases.*

|  | **Sequencing reads** | **Assembly size (sequences)** | **Assembly size (bases)** |
| --- | --- | --- | --- |
| Ml 1 | 17465080 | [73894](https://img.jgi.doe.gov/cgi-bin/mer/main.cgi?section=MetaDetail&page=scaffolds&taxon_oid=3300011239&data_type=assembled) | [33376241](https://img.jgi.doe.gov/cgi-bin/mer/main.cgi?section=MetaDetail&page=scaffolds&taxon_oid=3300011239&data_type=assembled) |
| Ml 2 | 28186722 | [66450](https://img.jgi.doe.gov/cgi-bin/mer/main.cgi?section=MetaDetail&page=scaffolds&taxon_oid=3300011237&data_type=assembled) | 32695412 |
| Ml 3 | 16801828 | [28727](https://img.jgi.doe.gov/cgi-bin/mer/main.cgi?section=MetaDetail&page=scaffolds&taxon_oid=3300011199&data_type=assembled) | [17783002](https://img.jgi.doe.gov/cgi-bin/mer/main.cgi?section=MetaDetail&page=scaffolds&taxon_oid=3300011199&data_type=assembled) |
| Ml 4 | 25805412 | [18484](https://img.jgi.doe.gov/cgi-bin/mer/main.cgi?section=MetaDetail&page=scaffolds&taxon_oid=3300011195&data_type=assembled) | 6405001 |
| Ml 5 | 20630402 | [25643](https://img.jgi.doe.gov/cgi-bin/mer/main.cgi?section=MetaDetail&page=scaffolds&taxon_oid=3300011197&data_type=assembled) | 10899365 |
| Ml 6 | 21421268 | [36952](https://img.jgi.doe.gov/cgi-bin/mer/main.cgi?section=MetaDetail&page=scaffolds&taxon_oid=3300011200&data_type=assembled) | 16375348 |
| Ml 7 | 22655970 | [26238](https://img.jgi.doe.gov/cgi-bin/mer/main.cgi?section=MetaDetail&page=scaffolds&taxon_oid=3300011198&data_type=assembled) | 13376228 |
| Ml 8 | 20003790 | [10239](https://img.jgi.doe.gov/cgi-bin/mer/main.cgi?section=MetaDetail&page=scaffolds&taxon_oid=3300011192&data_type=assembled) | 3947663 |
| Ml 9 | 22650764 | [7883](https://img.jgi.doe.gov/cgi-bin/mer/main.cgi?section=MetaDetail&page=listScaffolds&taxon_oid=3300011176&data_type=assembled) | 3544627 |
| Ml 10 | 58387594 | [52836](https://img.jgi.doe.gov/cgi-bin/mer/main.cgi?section=MetaDetail&page=scaffolds&taxon_oid=3300011227&data_type=assembled) | 25394805 |
| Ml 11 | 56518916 | [78995](https://img.jgi.doe.gov/cgi-bin/mer/main.cgi?section=MetaDetail&page=scaffolds&taxon_oid=3300011241&data_type=assembled) | 29749304 |
| Ml 12 | 37925220 | [15664](https://img.jgi.doe.gov/cgi-bin/mer/main.cgi?section=MetaDetail&page=scaffolds&taxon_oid=3300011193&data_type=assembled) | 4751816 |
| Ml 13 | 3952496 | 612 | 272071 |
| Ml 14 | 22179120 | [10999](https://img.jgi.doe.gov/cgi-bin/mer/main.cgi?section=MetaDetail&page=scaffolds&taxon_oid=3300011194&data_type=assembled) | 8951989 |
| Ml 15 | 6433798 | [608](https://img.jgi.doe.gov/cgi-bin/mer/main.cgi?section=MetaDetail&page=listScaffolds&taxon_oid=3300011172&data_type=assembled) | 241660 |
| Ml 16 | 22231192 | [6823](https://img.jgi.doe.gov/cgi-bin/mer/main.cgi?section=MetaDetail&page=listScaffolds&taxon_oid=3300011191&data_type=assembled) | 2827176 |
| Ml 17 | 38684672 | [18250](https://img.jgi.doe.gov/cgi-bin/mer/main.cgi?section=MetaDetail&page=scaffolds&taxon_oid=3300011196&data_type=assembled) | 16687388 |
| Ml 18 | 68392102 | [78312](https://img.jgi.doe.gov/cgi-bin/mer/main.cgi?section=MetaDetail&page=scaffolds&taxon_oid=3300011244&data_type=assembled) | 52131008 |
| Ml 19 | 23405212 | [3192](https://img.jgi.doe.gov/cgi-bin/mer/main.cgi?section=MetaDetail&page=listScaffolds&taxon_oid=3300011174&data_type=assembled) | 998864 |
| Ml 20 | 20152212 | [2871](https://img.jgi.doe.gov/cgi-bin/mer/main.cgi?section=MetaDetail&page=listScaffolds&taxon_oid=3300011173&data_type=assembled) | [859271](https://img.jgi.doe.gov/cgi-bin/mer/main.cgi?section=MetaDetail&page=listScaffolds&taxon_oid=3300011173&data_type=assembled) |
| Ml 21 | 28777734 | [6620](https://img.jgi.doe.gov/cgi-bin/mer/main.cgi?section=MetaDetail&page=listScaffolds&taxon_oid=3300011175&data_type=assembled) | 2019562 |
| Ml 22 | 22182332 | [115393](https://img.jgi.doe.gov/cgi-bin/mer/main.cgi?section=MetaDetail&page=scaffolds&taxon_oid=3300011246&data_type=assembled) | 61826702 |
| Ml 23 | 28234260 | [120239](https://img.jgi.doe.gov/cgi-bin/mer/main.cgi?section=MetaDetail&page=scaffolds&taxon_oid=3300011249&data_type=assembled) | 81857371 |
|  |  |  |  |
| Rl 1 | 67748804 | 204281 | 181720264 |
| Rl 2 | 77474456 | 205426 | 192219830 |
| Rl 3 | 61199512 | 176255 | 115011779 |
| Rl 4 | 74992750 | 314429 | 325818814 |
| Rl 5 | 74220220 | 374929 | 341656927 |
| Rl 6 | 68161082 | 292741 | 302935645 |
| Rl 7 | 75975046 | 274719 | 186606224 |
| Rl 8 | 91844214 | 588635 | 395690349 |
| Rl 9 | 77023868 | 238198 | 157627061 |
| Rl 10 | 139381778 | 344195 | 180851046 |
| Rl 11 | 91962344 | 203511 | 112190691 |
| Rl 12 | 81681122 | 86956 | 82284058 |
| Rl 13 | 105431412 | 251779 | 123396624 |
| Rl 14 | 63240678 | 37335 | 22184640 |
| Rl 15 | 72243790 | 125592 | 88672076 |
| Rl 16 | 82137112 | 232607 | 118301737 |
| Rl 17 | 107570706 | 393735 | 228570150 |
| Rl 18 | 78274268 | 162862 | 98312835 |
| Rl 19 | 75392190 | 99149 | 52942968 |
| Rl 20 | 65582044 | 81726 | 58507862 |
| Rl 21 | 91522052 | 264245 | 149668891 |
| Rl 22 | 96384726 | 236649 | 128282042 |
| Rl 23 | 96207238 | 406505 | 410018759 |
| Rl 24 | 80981820 | 99519 | 60773858 |
|  |  |  |  |
| Rb 1 | 76498938 | [350951](https://img.jgi.doe.gov/cgi-bin/mer/main.cgi?section=MetaDetail&page=scaffolds&taxon_oid=3300015024&data_type=assembled) | 429543524 |
| Rb 2 | 72691820 | [275858](https://img.jgi.doe.gov/cgi-bin/mer/main.cgi?section=MetaDetail&page=scaffolds&taxon_oid=3300015163&data_type=assembled) | 181694382 |
| Rb 3 | 70126632 | [334545](https://img.jgi.doe.gov/cgi-bin/mer/main.cgi?section=MetaDetail&page=scaffolds&taxon_oid=3300015194&data_type=assembled) | 249845086 |
| Rb 4 | 61828928 | [303591](https://img.jgi.doe.gov/cgi-bin/mer/main.cgi?section=MetaDetail&page=scaffolds&taxon_oid=3300015189&data_type=assembled) | 231705319 |
| Rb 5 | 74667258 | [375682](https://img.jgi.doe.gov/cgi-bin/mer/main.cgi?section=MetaDetail&page=scaffolds&taxon_oid=3300015193&data_type=assembled) | 213265070 |
|  |  |  |  |
| St 1 | 68111048 | [336424](https://img.jgi.doe.gov/cgi-bin/mer/main.cgi?section=MetaDetail&page=scaffolds&taxon_oid=3300015191&data_type=assembled) | 219250082 |
| St 2 | 71828498 | [154126](https://img.jgi.doe.gov/cgi-bin/mer/main.cgi?section=MetaDetail&page=scaffolds&taxon_oid=3300015078&data_type=assembled) | 83781202 |
| St 3 | 85214054 | [320463](https://img.jgi.doe.gov/cgi-bin/mer/main.cgi?section=MetaDetail&page=scaffolds&taxon_oid=3300015167&data_type=assembled) | 188269776 |
| St 4 | 71411294 | [184331](https://img.jgi.doe.gov/cgi-bin/mer/main.cgi?section=MetaDetail&page=scaffolds&taxon_oid=3300015082&data_type=assembled) | 103140952 |
| St 5 | 66910678 | 323872 | 236040965 |
| St 6 | 72683122 | 384288 | 290006175 |
| St 7 | 60148730 | 298633 | 216231874 |
| St 8 | 67844804 | 75868 | 48601571 |
| St 9 | 63953088 | 437238 | 275297528 |
| St 10 | 83010234 | [324248](https://img.jgi.doe.gov/cgi-bin/mer/main.cgi?section=MetaDetail&page=scaffolds&taxon_oid=3300015190&data_type=assembled) | 214964286 |
| St 11 | 74901072 | [263784](https://img.jgi.doe.gov/cgi-bin/mer/main.cgi?section=MetaDetail&page=scaffolds&taxon_oid=3300015164&data_type=assembled) | 180223385 |
| St 12 | 83405572 | [261266](https://img.jgi.doe.gov/cgi-bin/mer/main.cgi?section=MetaDetail&page=scaffolds&taxon_oid=3300015162&data_type=assembled) | 175258662 |
| St 13 | 64225756 | [179953](https://img.jgi.doe.gov/cgi-bin/mer/main.cgi?section=MetaDetail&page=scaffolds&taxon_oid=3300015084&data_type=assembled) | 121520140 |
| St 14 | 64764076 | [196033](https://img.jgi.doe.gov/cgi-bin/mer/main.cgi?section=MetaDetail&page=scaffolds&taxon_oid=3300015086&data_type=assembled) | 140418783 |
| St 15 | 66675200 | [146437](https://img.jgi.doe.gov/cgi-bin/mer/main.cgi?section=MetaDetail&page=scaffolds&taxon_oid=3300015076&data_type=assembled) | 83396680 |
| St 16 | 66848090 | [149134](https://img.jgi.doe.gov/cgi-bin/mer/main.cgi?section=MetaDetail&page=scaffolds&taxon_oid=3300015079&data_type=assembled) | 96714442 |
| St 17 | 72051286 | [332892](https://img.jgi.doe.gov/cgi-bin/mer/main.cgi?section=MetaDetail&page=scaffolds&taxon_oid=3300015195&data_type=assembled) | 268415228 |
| St 18 | 78397352 | [478826](https://img.jgi.doe.gov/cgi-bin/mer/main.cgi?section=MetaDetail&page=scaffolds&taxon_oid=3300015208&data_type=assembled) | 377436650 |

***Supplementary Figure 1:*** *Rarefaction curves for metagenomes sampled from Midtre Lovenbreen, Svalbard. The total assembled contigs in each metagenome is shown, against the total species count obtained from these contigs.*

***Supplementary Figure 2:*** *Rarefaction curves for metagenomes sampled from Russell Glacier, Greenland. The total assembled contigs in each metagenome is shown, against the total species count obtained from these contigs.*

***Supplementary Figure 3:*** *Rarefaction curves for metagenomes sampled from Storglaciaren, N-Sweden. The total assembled contigs in each metagenome is shown, against the total species count obtained from these contigs.*

***Supplementary Figure 4:*** *Rarefaction curves for metagenomes sampled from Rabots glacier, N-Sweden. The total assembled contigs in each metagenome is shown, against the total species count obtained.*

***Table S3:*** *GenBank and UniProtKB accession numbers for nifH sequences derived from Deslippe and Egger (2006), for use in the nifH phylogeny.*

| **Database** | **Accession number** | **Species** | **Gene** |
| --- | --- | --- | --- |
| GenBank  GenBank  GenBank  GenBank  GenBank  GenBank  UniProtKB  GenBank  GenBank  UniProtKB  UniProtKB  GenBank  GenBank  GenBank  UniProtKB  UniProtKB  GenBank  GenBank  GenBank  GenBank  UniProtKB  GenBank  UniProtKB  GenBank  GenBank | X13519.1  AY367395.1  AF484674.1  AF216883.1  V01215.1  U97122.1  P26251  AJ515294.1  Z31716.1  P33178  P08925  X57006.1  ABQ25379.1  M23528.1  P16269  Q07942  AF065617.1  AY221832.1  AF227926.1  AY040513.1  P25767  AY029234.1  P00456  AF065618.1  AF216881.1 | *Azotobacter vinelandii*  *Kiebsiella variicola*  *Methylomonas rubra*  *Azomonas agilis*  *Rhizobium meliloti*  *Azoarcus tolulyticus*  *Azorhizobium caulinodans*  *Paenibacillus azotofixans*  *Nostoc sp.*  *Anabaena sp.*  *Frankia alni*  *Frankia sp.*  *Geobacter uraniireducens*  *Azotobacter vinelandii*  *Azotobacter vinelandii*  *Azotobacter capsulatus*  *Chlorobium tepidum*  *Pelodictyon lutolum CC11OA0*  *Desulfovibrio salexigens*  *Desulfomicrobium baculatum*  *Methanococcus thermolithotrophicus*  *Methanosarcina mazei*  *Clostridium pasteurianum*  *Desulfonema limicola*  *Acetobacterium woodii* | vnfH  nifH  nifH  nifH  nifH  nifH  nifH  nifH  nifH  nifH  nifH  nifH  nifH  anfH  anfH  anfH  anfH  anfH  nifH  anfH  nifH  nifH  nifH  anfH  anfH |

***Table S4:*** *ANOVA comparing differences between the four forefields (Midtre Lovenbreen, Russell, Rabots and Storglaciaren) based on total nitrogen (TN) and total organic carbon (TOC). Significant differences observed between the forefields are noted at the 0.01 or 0.05 level.*

|  | **TN** | **TOC** |
| --- | --- | --- |
| **f-ratio value** | 2.46 | 5.375 |
| **p-value** | 0.071 | 0.002357 |
| **Significance level** | Not significant | 0.05 |

***Table S5:*** *Results of a post-Hoc Tukey analysis, comparing differences between forefields (Midtre Lovenbreen Ml, Russell Rl, Storglaciaren St and Rabots Rb), based on total nitrogen (TN) and total organic carbon (TOC). Significant differences between the forefields are noted at the 0.01 significance level.*

| **TN** | **Treatments** | **Q statistic** | **p-value** | **Inferfence** |
| --- | --- | --- | --- | --- |
|  | **Ml vs Rl** | 2.6457 | 0.2511852 | insignificant |
|  | **Ml vs Rb** | 0.1373 | 0.8999947 | insignificant |
|  | **Ml vs St** | 0.8928 | 0.8999947 | insignificant |
|  | **Rl vs Rb** | 1.5369 | 0.6773608 | insignificant |
|  | **Rl vs St** | 3.6426 | 0.0584388 | insignificant |
|  | **Rb vs St** | 0.7223 | 0.8999947 | insignificant |
|  |  |  |  |  |
| **TOC** | **Treatments** | **Q statistic** | **p-value** | **Inferfence** |
|  | **Ml vs Rl** | 3.5619 | 0.0669083 | insignificant |
|  | **Ml vs Rb** | 0.5212 | 0.8999947 | insignificant |
|  | **Ml vs St** | 1.6629 | 0.6279512 | insignificant |
|  | **Rl vs Rb** | 2.7952 | 0.2080416 | insignificant |
|  | **Rl vs St** | 5.3979 | 0.0017491 | ** p<0.01 |
|  | **Rb vs St** | 0.564 | 0.8999947 | insignificant |
|  |  |  |  |  |

***Table S6****: Initial and final concentrations of total nitrogen (TN) and total organic carbon (TOC) in forefield soils. Values are shown at the start of the transect/ chronosequence (by the glacier terminus) and at the end of the transect.*

|  | **Start TN (mg g^-1^)** | **End TN (mg g-1)** | **Start TOC (mg g^-1^)** | **End TN (mg g^-1^)** |
| --- | --- | --- | --- | --- |
| **Ml** | b.d | 4.40 | 2.85 | 14.47 |
| **St** | 0.21 | 0.40 | 0.46 | 1.24 |
| **Rb** | b.d | 1.74 | 0.29 | 0.70 |
| **Rl** | b.d | 1.33 | 0.13 | 17.46 |

***Figure S5:*** *rpoB normalized nif gene abundance and percentage of reads with an Alignment Score (AS) over 60, for samples obtained from Midtre Lovenbreen (Ml), Svalbard. The Alignment Score ranges between 0 and the maximum length of the reads (0-100) and indicates the quality of the alignment between reads and contigs.*

***Figure S6:*** *rpoB normalized nif gene abundance and percentage of reads with an Alignment Score (AS) over 60, for samples obtained from Russell Glacier (Rl), Greenland. The Alignment Score ranges between 0 and the maximum length of the reads (0-150) and indicates the quality of the alignment between reads and contigs.*

***Figure S7:*** *rpoB normalized nif gene abundance and percentage of reads with an Alignment Score (AS) over 60, for samples obtained from Rabots Glacier (Rb), N-Sweden. The Alignment Score ranges between 0 and the maximum length of the reads (0-150) and indicates the quality of the alignment between reads and contigs.*

***Figure S7:*** *rpoB normalized nif gene abundance and percentage of reads with an Alignment Score (AS) over 60, for samples obtained from Storglaciaren Glacier (St), N-Sweden. The Alignment Score ranges between 0 and the maximum length of the reads (0-150) and indicates the quality of the alignment between reads and contigs.*

***Table S7:*** *Mapping alignments between nif genes and raw sequencing reads. The number of read alignments in each metagenome is shown, grouped by the alignment score (AS) >=30, >=60, >=90, >=120 and >=140. A total for each forefield is also provided. The alignment score represents the quality of the alignment and ranges between 0-100 for the MI dataset and 0-150 for the Rb, St and RI datasets. The number of sequencing reads is provided, shown as the total number of forward and reverse reads for each sample. The percentage of alignments with an AS equal or higher than 60 is listed for each metagenome sample (% AS >= 60).*

| **Sample** | **Number of reads** | **AS >= 30** | **AS >= 60** | **AS >= 90** | **AS >= 120** | **AS >= 140** | **% AS >= 60** |
| --- | --- | --- | --- | --- | --- | --- | --- |
| **Ml 1** | 17465080 | 407 | 342 | 280 | - | - | 0.0019582 |
| **Ml 2** | 28186722 | 1175 | 1089 | 947 | - | - | 0.0038635 |
| **Ml 3** | 16801828 | 62 | 39 | 20 | - | - | 0.0002321 |
| **Ml 4** | 25805412 | 466 | 268 | 79 | - | - | 0.0010385 |
| **Ml 5** | 20630402 | 242 | 118 | 16 | - | - | 0.0005720 |
| **Ml 6** | 21421268 | 1653 | 1008 | 449 | - | - | 0.0047056 |
| **Ml 7** | 22655970 | 172 | 91 | 8 | - | - | 0.0004017 |
| **Ml 8** | 20003790 | 1040 | 542 | 69 | - | - | 0.0027095 |
| **Ml 9** | 22650764 | 344 | 175 | 17 | - | - | 0.0007726 |
| **Ml 10** | 58387594 | 2260 | 1336 | 230 | - | - | 0.0022882 |
| **Ml 11** | 56518916 | 244 | 135 | 30 | - | - | 0.0002389 |
| **Ml 12** | 37925220 | 314 | 161 | 22 | - | - | 0.0004245 |
| **Ml 13** | 3952496 | 72 | 38 | 3 | - | - | 0.0009614 |
| **Ml 14** | 22179120 | 660 | 355 | 46 | - | - | 0.0016006 |
| **Ml 15** | 6433798 | 164 | 93 | 11 | - | - | 0.0014455 |
| **Ml 16** | 22231192 | 125 | 68 | 8 | - | - | 0.0003059 |
| **Ml 17** | 38684672 | 333 | 197 | 31 | - | - | 0.0005092 |
| **Ml 18** | 68392102 | 932 | 560 | 93 | - | - | 0.0008188 |
| **Ml 19** | 23405212 | 79 | 40 | 6 | - | - | 0.0001709 |
| **Ml 20** | 20152212 | 37 | 20 | 8 | - | - | 0.0000992 |
| **Ml 21** | 28777734 | 98 | 61 | 12 | - | - | 0.0002120 |
| **Ml 22** | 22182332 | 24 | 4 | 0 | - | - | 0.0000180 |
| **Ml 23** | 28234260 | 6 | 0 | 0 | - | - | 0.0000000 |
| **Ml forefield** | 633078096 | 10909 | 6740 | 2385 | 0 | 0 | 0.0010646 |
| **Rl 1** | 67748804 | 28 | 0 | 0 | 0 | 0 | 0.0000000 |
| **Rl 2** | 77474456 | 20 | 0 | 0 | 0 | 0 | 0.0000000 |
| **Rl 3** | 61199512 | 39 | 11 | 7 | 0 | 4 | 0.0000180 |
| **Rl 4** | 74992750 | 130 | 80 | 64 | 23 | 49 | 0.0001067 |
| **Rl 5** | 74220220 | 109 | 60 | 43 | 16 | 34 | 0.0000808 |
| **Rl 6** | 68161082 | 240 | 156 | 121 | 46 | 90 | 0.0002289 |
| **Rl 7** | 75975046 | 28 | 0 | 0 | 0 | 0 | 0.0000000 |
| **Rl 8** | 91844214 | 47 | 0 | 0 | 0 | 0 | 0.0000000 |
| **Rl 9** | 77023868 | 34 | 6 | 4 | 2 | 3 | 0.0000078 |
| **Rl 10** | 139381778 | 32 | 5 | 1 | 0 | 0 | 0.0000036 |
| **Rl 11** | 91962344 | 131 | 63 | 32 | 16 | 27 | 0.0000685 |
| **Rl 12** | 81681122 | 61 | 29 | 18 | 1 | 9 | 0.0000355 |
| **Rl 13** | 105431412 | 190 | 143 | 92 | 18 | 53 | 0.0001356 |
| **Rl 14** | 63240678 | 26 | 10 | 4 | 0 | 3 | 0.0000158 |
| **Rl 15** | 72243790 | 428 | 215 | 76 | 5 | 29 | 0.0002976 |
| **Rl 16** | 82137112 | 23 | 6 | 1 | 0 | 0 | 0.0000073 |
| **Rl 17** | 107570706 | 74 | 24 | 10 | 4 | 6 | 0.0000223 |
| **Rl 18** | 78274268 | 33 | 3 | 1 | 0 | 1 | 0.0000038 |
| **Rl 19** | 75392190 | 30 | 10 | 2 | 0 | 0 | 0.0000133 |
| **Rl 20** | 65582044 | 16 | 0 | 0 | 0 | 0 | 0.0000000 |
| **Rl 21** | 91522052 | 24 | 1 | 0 | 0 | 0 | 0.0000011 |
| **Rl 22** | 96384726 | 43 | 14 | 3 | 0 | 2 | 0.0000145 |
| **Rl 23** | 96207238 | 5 | 0 | 0 | 0 | 0 | 0.0000000 |
| **Rl 24** | 80981820 | 30 | 9 | 3 | 0 | 1 | 0.0000111 |
| **Rl forefield** | 1996633232 | 1821 | 845 | 482 | 131 | 311 | 0.0000423 |
| **Rb 1** | 76498938 | 95 | 29 | 23 | 2 | 11 | 0.0000379 |
| **Rb 2** | 72691820 | 211 | 117 | 76 | 21 | 49 | 0.0001610 |
| **Rb 3** | 70126632 | 379 | 199 | 132 | 55 | 104 | 0.0002838 |
| **Rb 4** | 61828928 | 584 | 396 | 307 | 99 | 226 | 0.0006405 |
| **Rb 5** | 74667258 | 215 | 105 | 52 | 10 | 32 | 0.0001406 |
| **Rb Forefield** | 355813576 | 1484 | 846 | 590 | 187 | 422 | 0.0002378 |
| **St 1** | 68111048 | 247 | 120 | 67 | 8 | 37 | 0.0001762 |
| **St 2** | 71828498 | 1817 | 1385 | 1031 | 480 | 749 | 0.0019282 |
| **St 3** | 85214054 | 2871 | 2235 | 1571 | 734 | 1143 | 0.0026228 |
| **St 4** | 71411294 | 1967 | 1552 | 1150 | 484 | 804 | 0.0021733 |
| **St 5** | 66910678 | 726 | 563 | 457 | 285 | 389 | 0.0008414 |
| **St 6** | 72683122 | 142 | 59 | 39 | 17 | 28 | 0.0000812 |
| **St 7** | 60148730 | 645 | 481 | 349 | 190 | 274 | 0.0007997 |
| **St 8** | 67844804 | 1956 | 1586 | 1213 | 784 | 1026 | 0.0023377 |
| **St 9** | 63953088 | 1447 | 1222 | 1003 | 616 | 851 | 0.0019108 |
| **St 10** | 83010234 | 102 | 60 | 44 | 25 | 38 | 0.0000723 |
| **St 11** | 74901072 | 577 | 459 | 369 | 182 | 297 | 0.0006128 |
| **St 12** | 83405572 | 586 | 384 | 259 | 100 | 182 | 0.0004604 |
| **St 13** | 64225756 | 119 | 86 | 71 | 19 | 44 | 0.0001339 |
| **St 14** | 64764076 | 295 | 229 | 182 | 83 | 140 | 0.0003536 |
| **St 15** | 66675200 | 350 | 238 | 185 | 101 | 150 | 0.0003570 |
| **St 16** | 66848090 | 363 | 302 | 250 | 127 | 203 | 0.0004518 |
| **St 17** | 72051286 | 1064 | 947 | 760 | 447 | 611 | 0.0013143 |
| **St 18** | 78397352 | 485 | 346 | 260 | 118 | 209 | 0.0004413 |
| **St Forefield** | 1282383954 | 15759 | 12254 | 9260 | 4800 | 7175 | 0.0009556 |

***Table S8****: NCBI blastn matches for sample sequences, against cultured isolates. The best match accession number and % identity is given.* *Sequences with no significant matches have been left blank.*

| **Sample** | **Cluster** | **Blast match** | **Accession number** | **% similarity** |
| --- | --- | --- | --- | --- |
| Rb10 | I | *Frankia sp.* | X57006.1 | 83 |
| Ml 10 | I | *Frankia sp.* | CP000820.1 | 84 |
| Ml 6 | I | *Frankia alni str.* | CT573213.2 | 82 |
| Ml 18 | I | *-* | - | - |
| Ml 10 | I | *Frankia casuarinae strain* | CP000249.1 | 82 |
| St 11 | I | *Frankia sp.* | AY115490.2 | 82 |
| Ml 2 | I | *Frankia alni str.* | CT573213.2 | 82 |
| St 17 | I | *Frankia HRN18a* | X17522.1 | 81 |
| St 8 | I | *Frankia sp.* | X73983.1 | 81 |
| St 17 | I | *Frankia sp.* | HM026362.1 | 81 |
| Rl13 | I | *Leptosprillum ferriphilum* | JN390678.1 | 85 |
|  |  |  |  |  |
| St3 | III | *Geobacter uraniireducens* | CP000698.1 | 88 |
| St3 | III | *Geobacter lovleyi* | CP001089.1 | 90 |
| St5 | III | *Geobacter uraniireducens* | CP000698.1 | 89 |
|  |  |  |  |  |
| Rl 6 | I | *Nostoc flagelliforme* | AP018269.1 | 95 |
| Rl 6 | I | *Scytonema sp.* | AP018268.1 | 91 |
| Ml1 | I | *Nostoc punctiforme* | CP001037.1 | 94 |
| Ml1 | I | *Scytonema sp.* | AP018268.1 | 90 |
| St15 | I | *Scytonema sp.* | AP018268.1 | 84 |
| St9 | I | *Anabaena variabilis* | AP018216.1 | 84 |
|  |  |  |  |  |
| Rb2 | I | *Bradyrhizobium oligotrophicum* | AP012603.1 | 94 |
| Rb3 | I | *Bradyrhizobium oligotrophicum* | AP012603.1 | 91 |
| St 11 | I | *Polaromonas napthalenivorans CJ2* | CP000529.1 | 87 |
|  |  |  |  |  |
| St18 | I | *Bradyrhizobium oligotrophicum S58* | AP012603.1 | 80 |
| St11 | I | *Polaramonas napthalenivorans CJ2* | CP000529.1 | 94 |
| St11 | I | *Polaromonas napthalenivorans CJ2* | CP000529.1 | 90 |
